# Humoral and Cellular Responses to BNT162b2 as a Booster Following Two Doses of ChAdOx1 nCov-19 Determined Using Three SARS-CoV-2 Antibody Assays and an Interferon-Gamma Release Assay: A Prospective Longitudinal Study in Healthcare Workers

**DOI:** 10.3389/fimmu.2022.859019

**Published:** 2022-06-01

**Authors:** Seri Jeong, Nuri Lee, Su Kyung Lee, Eun-Jung Cho, Jungwon Hyun, Min-Jeong Park, Wonkeun Song, Hyun Soo Kim

**Affiliations:** ^1^ Department of Laboratory Medicine, Hallym University Kangnam Sacred Heart Hospital, Hallym University College of Medicine, Seoul, South Korea; ^2^ Department of Laboratory Medicine, Hallym University Dongtan Sacred Heart Hospital, Hallym University College of Medicine, Gyeonggi-do, South Korea

**Keywords:** SARS-CoV-2, BNT162b2, ChAdOx1 nCov-19, booster, vaccine, antibody, assay, INF-γ

## Abstract

Data on humoral and cellular responses to BNT162b2 as a booster dose, following two doses of ChAdOx1 nCov-19 vaccine, have seldom been reported. The aim of this study was to assess the positivity rates of three representative antibody assays targeting total, IgG, and neutralizing antibodies, and an interferon-γ release assay (IGRA), and to determine the longitudinal changes in quantitative antibody titers after each vaccination. A total of 1027 samples were collected from healthcare workers. The number of participants after the booster dose was 153, and they all completed a questionnaire on adverse reactions. All antibody assays showed 100.0% positivity at 1 month after booster vaccination. The median antibody titers of the assays were significantly increased compared with those after the second dose (22.1-fold increase for Roche total antibody, 14.0-fold increase for Abbott IgG, and 1.1-fold increase (97.5% inhibition) for GenScript neutralizing antibody). Cellular responses determined using the IGRA were positive in 92.8% of the participants. Most participants (72.5%) reported mild adverse reactions. Correlations between the three antibody assays and IGRA were weak or negligible, indicating a difference between humoral and cellular responses. Overall, our study provides information about booster vaccine strategies and laboratory settings, which could subsequently contribute to the control of the spread of coronavirus disease 2019.

## Introduction

The coronavirus disease 2019 (COVID-19), caused by severe acute respiratory syndrome coronavirus 2 (SARS-CoV-2), has seriously affected global public health. The World Health Organization (WHO) has reported 290,959,019 confirmed cases of COVID-19 and 5,446,753 deaths worldwide until January 5, 2022 (1). Universal vaccination has been adopted as the most effective strategy to alleviate COVID-19 severity and prevent deaths, despite the substantial and rapid spread of variants (2–4). Various types of vaccines based on mRNA, viral vectors, and inactivated viruses have been developed (5, 6) and administered to date, amounting to 8,693,832,171 doses globally (1). Although most studies have demonstrated good protective effect of the initial vaccination regimen (6), the Joint Committee on Vaccination and Immunization has recommended a booster dose 6 months after the completion of primary vaccinations owing to the emerging evidence of waning protective effect of the vaccines (7). To disseminate information about the optimal booster vaccine, the UK COV-BOOST trial reported the immunogenicity and safety of seven representative vaccines administered as a booster following the complete doses of ChAdOx1 nCoV-19 or BNT162b2. Booster vaccines have been found to be effective in terms of substantially increased responses of anti-spike IgG and neutralizing antibodies, with no safety concerns, in participants older than 30 years of age (8).

The importance of humoral immunity for protection against COVID-19 has been demonstrated (9, 10). Various assays based on enzyme-linked immunosorbent assay (ELISA) or chemiluminescence immunoassay have been developed and applied to measure anti-SARS-CoV-2 antibodies (11, 12). Some commercially available assays targeting total antibodies and IgG have exhibited high analytical performance (13, 14), and they are frequently used in studies for determining and monitoring immune responses after vaccination. The pseudovirus neutralization assay, surrogate virus neutralization test (sVNT), and plaque reduction neutralization test have been widely applied to determine neutralizing antibodies with protective functions (8, 11, 15). In particular, the recently introduced sVNT kit can be used to measure the blockage between angiotensin-converting enzyme 2 (ACE2) and receptor-binding domain (RBD); it exhibits similar or improved performance compared to the live-cell neutralization test. In addition, its rapidity and easy accessibility with low biological hazards make it preferable over other test (15).

T cell-mediated immunity plays an important role in controlling COVID-19 severity and rapid viral clearance (16). Furthermore, the reactivity of cellular immunity to SARS-CoV-2 variants has been found persist even if the viral cells escape humoral immunity (17, 18). Heterologous vaccination is reportedly associated with higher cellular responses than homologous schedules (19). Cellular responses have been measured using various methods (8, 19). SARS-CoV-2-specific interferon-γ (IFN-γ) release assay, based on a methodology similar to that utilized for tuberculosis blood tests, has been introduced to determine cellular immunity, implicating sustained responses after vaccination (20, 21).

Data on the effects of booster vaccination after two doses of primary vaccines have been scarcely reported, although they would be important for establishing vaccine strategies. The aim of the current study was to investigate the humoral and cellular responses to BNT162b2, as a third dose following two complete doses of ChAdOx1 nCov-19, using the representative antibody assays targeting total, IgG, and neutralizing antibodies and the IFN-γ release assay in healthcare workers, including those in their 20s. Agreement among the antibody assays and their correlation with cellular responses were investigated to gain a better insight into the assays and provide information for improved laboratory settings.

## Materials and Methods

### Study Population and Samples

To study the serologic responses after BNT162b2 as a booster, following two complete doses of ChAdOx1 nCov-19, 153 healthcare workers of two university hospitals were enrolled. The inclusion criteria were age > 18 years and administration of two intramuscular injections of ChAdOx1 nCov-19 with a 12-week interval. After collecting 228 baseline samples, 1st and 2nd doses of ChAdOx1 nCov-19 and a booster dose of BNT162b2 were administered in March, May, and December 2021, respectively. Serum samples were collected from the workers to measure the presence of SARS-CoV-2 antibodies 1 month after each vaccination (n = 228 after the first dose and n = 218 after the second dose). Seven months after the first ChAdOx1 nCov-19 dose, 200 samples were collected to evaluate the longevity of antibodies against SARS-CoV-2. Data of these samples, published in our previous study (11, 22), have been deposited in a public database (https://doi.org/10.7910/DVN/HNDD7L for baseline and 1 month after the first dose; https://doi.org/10.7910/DVN/HPPSBA for 1 month after the second dose; and https://doi.org/10.7910/DVN/BXTUIR for 7 months after the first dose); they were extracted for this study. After initial sampling for baseline level, 75 healthcare workers were excluded owing to their resignation, refusal of additional vaccine injection or blood sampling, or injection of other types of vaccines, such as BNT162b2 as a second dose or mRNA-1273 as a booster. Finally, 153 workers remained, and their serum samples were obtained using a serum separator and two heparin tubes. All workers included in this study received a questionnaire on adverse reactions after the vaccination; the questionnaire after the booster BNT162b2 vaccination included questions regarding the order of severity after each vaccination, including the presence, severity, and duration of adverse reactions, and the use of antipyretics after the booster vaccination. All participants reported that there was no breakthrough infection during the study.

### Assays for Total, IgG, and Neutralizing Antibodies to SARS-CoV-2

Three commonly used SARS-CoV-2 antibody assays targeting total, IgG, and neutralizing antibodies were used to measure the serologic responses after the booster vaccination. For the total antibody, the Elecsys Anti-SARS-CoV-2 S assay on the Elecsys Cobas e801 platform (Roche Diagnostics, Mannheim, Germany), targeting the RBD, was utilized; the electrochemiluminescence immunoassay was based on the double-antigen sandwich principle. The required sample volume was 12 µL and the cutoff was 0.8 U/mL; it required 18 min to obtain the antibody levels. To determine the level of IgG targeting the RBD, SARS-CoV-2 IgG II Quant on Alinity I (Abbott, Abbott Park, IL, USA), a chemiluminescent microparticle immunoassay, was used. Its cutoff was 50 AU/mL, and the required sample volume and time were 25 µL and 29 min, respectively. The cPass SARS-CoV-2 Neutralization Antibody Detection kit (GenScript, Piscataway, NJ, USA), an sVNT, was used to determine RBD-binding neutralizing antibodies based on a competitive ELISA. Horseradish peroxidase-labeled RBD reagent and ACE2-coated ELISA plate were used for the purpose. When 10 µL of serum samples was loaded, the results for neutralizing antibodies were obtained after 80 min with a 30% (percent inhibition value) cutoff. An Epoch Microplate Spectrophotometer (BioTek Instruments, Winooski, VT, USA) and ELx50 Filter Microplate Washer (BioTek Instruments) were used for ELISA. All included assays were conducted according to the manufacturers’ instructions.

### Assay for Cellular Response to SARS-CoV-2

The Covi-FERON ELISA kit (SD Biosensor, Suwon, Korea) was used to detect cell-mediated immune responses to SARS-CoV-2 antigens by measuring IFN-γ secreted by T cells in response to the SARS-CoV-2 antigen (21). Blood samples collected in heparinized tubes were aliquoted (in 1 mL aliquots) into five tubes for Nil, Mitogen, original spike protein (SP1) antigen, variant spike protein antigen (SP2), and nucleocapsid protein (NP) antigens. The original SP1 antigen tube contained a spike protein derived from Wuhan/UK variant (B.1.1.7), whereas the variant SP2 antigen tube contained the spike protein derived from South Africa (B.1.351) and Brazil (P.1) variants. At least one positivity for either the original SP1 or variant SP2 assay was considered a positive result for this assay. Mitogen tubes were used as positive controls to assess the participants’ immune status. The Nil tubes were utilized as a negative control to adjust the background noise of INF-γ. Tubes with 1 mL of heparinized blood were incubated at 37°C for 16–24 h. Following the incubation period, the samples were centrifuged and the level of INF-γ in each tube was measured using an ELISA kit. Fifty microliters of supernatant in each tube was dispensed into the ELISA plate and mixed well. After incubation at 37°C for 1 h, the samples were washed five times with 350 µL of diluted wash buffer. TMB substrate was added to each well and incubated for 30 min at room temperature. After stopping the reaction, the absorbance of samples in the wells at 450 nm was measured. INF-γ concentration in each tube was calculated from the concentrations of four standard tubes and Nil tubes using ELISA software provided by SD Biosensor with a cutoff value of 0.25 IU/mL.

### Statistical Analysis

Chi-square tests for nominal variables were applied to the descriptive statistics. Dwass-Steel-Critchlow-Finger test for multiple comparisons was utilized to compare the longitudinal changes in anti-SARS-CoV-2 antibody levels among sampling times from baseline to booster of BNT162b2. Agreements among the total, IgG, and neutralizing antibody assays were assessed based on Cohen’s kappa values using the same categories as in a previous report (11). Briefly, 0.61–0.80 was designated as substantial and 0.81–1.00 was interpreted as almost perfect. Spearman’s rank correlation coefficients were used to evaluate the correlations among all included assays. The values less than 0.1 were negligible, and those between 0.1 and 0.39 were weak; coefficients of 0.70 to 0.89 were interpreted as strong. MedCalc software version 19.8 (MedCalc Software Ltd., Ostend, Belgium) and Analyse-it Method Evaluation Edition software version 2.26 (Analyse-it Software Ltd., Leeds, UK) were used for the analyses.

## Results

### Basic Characteristics of the Participants

The basic characteristics of all 153 healthcare workers enrolled after BNT162b booster injection following two doses of ChAdOx1 nCov-19 are summarized in **Table 1**. Most of them were women (83.0%), and their median age was 36.0 years, ranging from 22 to 60 years. Our study population mostly consisted of nurses (65.4%), followed by laboratory technicians (25.5%). The median number of sampling days after the booster dose was 24.0 days (1st to 3rd quartile range, 21.0–26.1 days). This corresponded to the median number of sampling days after the first dose of ChAdOx1 nCov-19 at 284.0 days (1st to 3rd quartile range, 280.0–285.0 days) and that of the second dose at 204.0 days (1st to 3rd quartile range, 201.9–207.0 days). As this was a longitudinal study, the number and characteristics of the participants, including the questionnaire after the 1st and 2nd doses of ChAdOx1 nCov-19, according to sampling time, are shown in **Table 1** using the data of previous studies (11, 22).

**Table 1:** Basic characteristics of and adverse reactions after vaccination in participants in this longitudinal study over a 10-month period.

Characteristic	Baseline (before vaccination) (*n* = 228)	After first ChAdOx1 nCoV-19 (*n* = 228)	After second ChAdOx1 nCoV-19 (*n* = 218)	Seven months after first ChAdOx1 nCoV-19 (*n* = 200)	After BNT162b2 booster (*n* = 153)
Age (n)					
21–30 y	101	101	95	80	57
31–40 y	50	50	48	46	35
41–50 y	46	46	44	44	35
51–60 y	31	31	31	30	26
Sex (n)					
Male	36	36	34	32	26
Female	192	192	184	168	127
Occupation (n)					
Nurse	154	154	147	130	100
Laboratory technician	58	58	57	54	39
Doctor	14	14	14	14	13
Others	2	2		2	1
Adverse reaction after booster (n)					
Absent		8	108		40
Mild		152	108		111
Severe		68	2		2
Duration of adverse reaction (n)					
0–1 d		67	171		70
2–3 d		141	39		68
>4 d		20	8		15
Order of severity of adverse reaction (1st dose, 2nd dose, and booster) (n)					
0-0-0^a^					6
0-0-1^a^					7
0-1-0^a^					1
0-1-2^a^					1
1-0-0^a^					26
1-0-2^a^					34
1-2-0^a^				7
1-2-3					26
1-3-2					24
2-0-1^a^					9
2-1-3					1
2-3-1					8
3-1-2					1
3-2-1					2
Antipyretics (n)					
Not taken		23	115		52
Taken		205			
Prophylactically taken			60		37
Taken with symptoms			43		64

a 0, no symptoms.

### Longitudinal Antibody Response Determined Using the SARS-CoV-2 Antibody Assays

**Figure 1:**
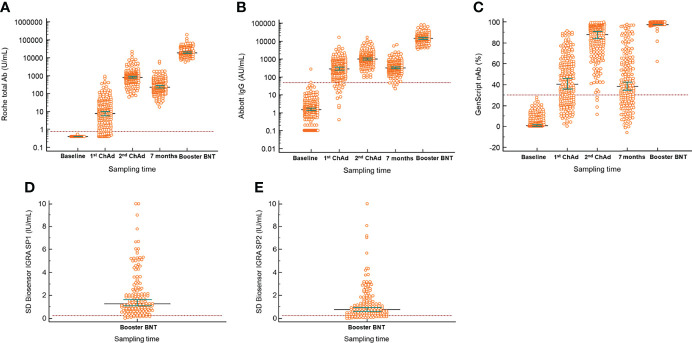
Longitudinal serological responses to BNT162b vaccine as a booster after two complete doses of ChAdOx1 nCov-19. **(A)** Roche total antibody; **(B)** Abbott IgG; **(C)** GenScript neutralizing antibody; **(D)** SD Biosensor interferon-gamma release assay of Original spike protein (SP1) tube; **(E)** SD Biosensor interferon-gamma release assay of Variant spike protein (SP2) tube. The difference between the first or second vaccination and booster injection in all included antibody assays was significant (*P* < 0.001). Horizontal lines and whiskers indicate median and 95% confidence interval. The dotted lines indicate the cut-off values of the assays. ChAd, ChAdOx1 nCoV-19; BNT, BNT162b2; IGRA, interferon-gamma release assay.

The quantitative antibody levels in the samples at different times are presented in **Table 2**. The median values of Roche total (18993.0 U/mL), Abbott IgG (14573.2 AU/mL), and GenScript neutralizing antibodies (97.5%) in the samples after the BNT162b2 booster vaccination were the highest among all those in the included timings, as depicted in **Figure 1**. Compared with the median values after the second ChAdOx1 nCoV-19 dose, significantly elevated antibody titers were observed after the booster dose of BNT162b2 (*P* < 0.001), suggesting an increase of 22.1-fold for Roche total antibody, 14.0-fold for Abbott IgG, and 1.1-fold (97.5% inhibition) for GenScript neutralizing antibody. All participants showed 100.0% positivity for Roche total, Abbott IgG, and GenScript neutralizing antibody assays when the cutoffs provided by the manufacturers were considered.

**Table 2:** Results of the anti-SARS-CoV-2 antibody assays and interferon-gamma releasing assay after two doses of ChAdOx1 nCoV-19 and a booster dose of BNT162b2.

Sampling time	Roche Total antibody		Abbott IgG		GenScript nAb		SD Biosensor IGRA		
	Titer (U/mL)^a^	Positivity (%)	Titer (AU/mL)^a^	Positivity (%)	Titer (%)^a^	Positivity (%)	Original (SP1) tubeTiter (IU/mL)^a^	Variant (SP2) tubeTiter (IU/mL)^a^	Positivity (%)
Baseline (n = 228)	<0.4	0	1.5 (0.5-3.3)	0.4	0.7 (0.1-7.5)	0	N.D.	N.D.	
After first ChAd (n = 228)	8.0 (1.7-26.7)	84.6	278.4 (114.3-732.8)	92.5	40.6 (24.4-59.5)	66.2	N.D.	N.D.	
After second ChAd (n = 218)	860.5 (485.3-1286.5)	100	1041.5 (631.5-1681.9)	100	88.5 (74.2-95.4)	98.2	N.D.	N.D.	
Seven months from first ChAd (n = 200)	232.0 (138.0-469.8)	100	325.5 (184.8-599.7)	97.0	38.2 (24.0-61.4)	66.0	N.D.	N.D.	
After BNT booster (n = 153)	18993.0 (14206.3-35850.0)	100	14573.2 (9673.4-22133.9)	100	97.5 (97.3-97.6)	100	1.275 (0.733-2.616),	0.7730 (0.380-1.688)	92.8
*P* ^b^	<0.001		<0.001		<0.001				

a Data are expressed as median (1st to 3rd quartiles).

b P values between the booster of BNT162b2 and the other doses.

nAb, neutralizing antibody.

IGRA, interferon-gamma releasing assay.

N.D. not done.

ChAd, ChAdOx1 nCoV-19.

BNT, BNT162b2.

### Cellular Response Determined Using Covi-FERON

Positivity for the Covi-FERON INF-γ response assay, related to cell-mediated immunity against SARS-CoV-2 antigens, was 92.8% at 1 month after the booster vaccination. The median value of IFN-γ in the SP1 tube was 1.3 IU/mL (1st to 3rd quartile range, 0.7–2.6 IU/mL), and that in the SP2 tube was 0.8 IU/mL (1st to 3rd quartile range, 0.4–1.7 IU/mL). The results in the NP tube were all negative.

### Adverse Reactions After the Booster Vaccination

The profiles of adverse reactions after vaccination with BNT162b2 as a booster are shown in **Table 1**. Most participants (98.7%) experienced mild or no adverse reactions. Myalgia was the most common symptom (54.1%), followed by a fever and feverish symptoms (30.3%). Among the participants with adverse reactions, most participants (90.1%) answered that the duration of adverse reactions was less than 3 days, and most (98.7%) reported that they had no symptoms or mild symptoms. In terms of antipyretics, 66.0% of the participants took drugs such as Tylenol tablets, provided at the vaccine administration site, and 36.6% took antipyretics prophylactically. The order of severity of adverse reactions after the 1st and 2nd doses of ChAdOx1 nCov-19 and the booster dose of BNT162b2 were investigated next, and most participants (76.4%) reported that the adverse reactions were the most severe after the first dose of ChAdOx1 nCov-19 among all three doses of vaccines.

### Agreement Among the SARS-CoV-2 Antibody Assays and the Correlation With Covi-FERON

**Figure 2:**
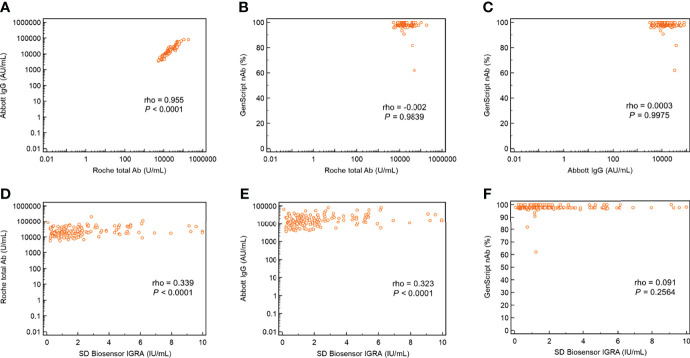
Correlation plots with ρ values of the three SARS-CoV-2 antibody assays and interferon-gamma release assay for cellular response following BNT162b booster vaccination. **(A)** Abbott IgG versus Roche total antibody; **(B)** GenScript neutralizing antibody versus Roche total antibody; **(C)** GenScript neutralizing antibody versus Abbott IgG; **(D)** Roche total antibody versus SD Biosensor interferon-gamma release assay; **(E)** Abbott IgG versus SD Biosensor interferon-gamma release assay; and **(F)** GenScript neutralizing antibody versus SD Biosensor interferon-gamma release assay.

The agreement rates among the three SARS-CoV-2 antibody assays using 1027 samples collected from healthcare workers (n = 228 for baseline, n = 228 after the first dose, n = 218 after the second dose, n = 200 at 7 months after the first vaccination, and n = 153 after the booster injection) are shown in **Table 3**. The total agreement rate for all 1027 samples was the highest (97.0%) between the Roche total and Abbott IgG antibody assays, and the kappa value was almost perfect (0.919). The agreement rate among the three SARS-CoV-2 antibody assays after the booster vaccination was 100%, as all samples showed positive results after the booster vaccination. The agreement rate between GenScript nAb and Roche total/Abbott IgG after the first ChAdOx1 nCoV-2 vaccination (78.9%/73.7%) and 7 months thereafter (66.3%/69.3%) was lower than that after the second dose (98.2%/98.2%) and booster dose (100%/100%). As illustrated in **Figure 2**, the correlation between the Roche total and Abbott IgG SARS-CoV-2 antibody assays after the booster vaccination was strong and showed the highest value (ρ = 0.955). The correlations of Roche total/Abbott IgG with GenScript nAb were weak, as GenScript nAb showed positive results of >90% after the booster vaccination. Correlations between the three antibody assays and SD Biosensor IGRA showed were weak or negligible.

**Table 3:** Agreement rates among four SARS-CoV-2 assays *^a^*.

A/B	Baseline (n = 228)	After the first ChAdOx1 nCoV-19 (n = 228)	After the second ChAdOx1 nCoV-19 (n = 218)	Seven months after the first ChAdOx1 nCoV-19 (n = 200)	After BNT162b2 booster (n = 153)	Total (n = 1027)	
						Agreement rate	Kappa value
Roche total/Abbott IgG	99.6 (97.6-99.9)	89.5 (84.7-93.1)	100	97.0 (93.6-98.9)	100	97.0 (95.7-97.9)	0.919
Roche total/GenScript nAb	100	78.9 (73.1-84.1)	98.2 (95.4-99.5)	66.3 (59.3-72.9)	100	88.4 (86.3-90.3)	0.732
Abbott IgG/GenScript nAb	99.6 (97.5-99.9)	73.7 (67.5-79.3)	98.2 (95.4-99.5)	69.3 (62.4-75.7)	100	87.7 (85.5-89.6)	0.714
Roche total/SD Biosensor IGRA	–	–	–	–	92.8 (87.5-96.4)	–	–
Abbott IgG/SD Biosensor IGRA	–	–	–	–	92.8 (87.5-96.4)	–	–
GenScript nAb/SD Biosensor IGRA	–	–	–	–	92.8 (87.5-96.4)	–	–

a Agreement rates are expressed as % (95% confidence interval), kappa value.

nAb, neutralizing antibody.

## Discussion

In this study, we investigated humoral and cellular responses to a booster dose of BNT162b after two doses of ChAdOx1 nCoV-19 in healthcare workers. Three SARS-CoV-2 assays for total, IgG, and neutralizing antibodies, and an IFN-γ release assay were conducted to determine the effectiveness of the booster injection. Our results showed a 100% positivity rate in the antibody assays; 95.1% reactivity was determined using Covi-FERON IGRA. Most participants (78.7%) experienced mild adverse reactions after the booster vaccination. Agreement among the three antibody assays was perfect (100%), but that with Covi-FERON was slightly low (92.8%). Correlations among the three antibody assays were strong, whereas those of the assays with Covi-FERON were weak or negligible, implying a difference between humoral and cellular responses.

It has been shown that cellular immunity was detectable at 8 months after two doses of BNT162b2, although antibody levels declined significantly (20). A study investigating the immunogenicity of 23 healthcare workers after the first dose of BNT162b2 or ChAdOx1 nCov-19 presented 67.7% positivity determined using Covi-FERON IGRA (21). The median values for BNT162b2 and ChAdOx1 nCov-19 were 1.656 and 0.978 IU/mL, respectively. The positivity (92.8%) and median levels (1.3 IU/mL) in our study were slightly higher than those reported previously (21, 23). The median value of variant SP2 tube (0.8 IU/mL) was lower than that of the original SP1 tube. Despite the lowered values, T cell responses to variant strains were sustained in vaccinated individuals, similar to that reported in a previous study (24).

Administration of a booster dose of BNT162b2 after two doses of BNT162b2 has been approved in Israel to address the reduced effectiveness of vaccine against variant strains and potential waning immunity over time. The third dose of BNT162b2 mRNA vaccine after two homogeneous primary vaccinations of BNT162b2 was found to be effective against severe COVID-19-related outcomes (25, 26). Research on the combination of several types of vaccines is considered important, as other types of vaccines such as ChAdOx1 nCoV-19 and mRNA-1273 have been administered in several countries according to the supply status and recipient preference. The mixed schedule of vaccines resulted in greater protection than that of homogeneous vaccinations. The UK Com-COV trial confirmed that the anti-spike IgG levels from heterologous schedules with ChAdOx1 nCoV-19 and BNT162b2 were approximately 9-fold higher than those from the homologous vaccine schedule with two ChAdOx1 nCoV-19 doses (19). For the combination of ChAdOx1 nCoV-19 and mRNA-1273, the UK Com-COV2 trial showed an approximately 10-fold increase in anti-spike IgG after heterologous second dosing with mRNA-1273 compared with homologous dosing of ChAdOx1 nCoV-19 (27). Our results for BNT162b2 injection as a booster following two doses of ChAdOx1 nCoV-19 revealed significantly increased median values of total IgG and neutralizing antibodies. This is in accordance with the results of the UK COV-BOOST trial reporting 24.48 geometric mean ratio for anti-spike IgG response in 95 participants compared with that in the control group. The study population of the UK COV-BOOST trial was composed of more than 90% Whites, and only individuals older than 30 years were enrolled. In addition, the median intervals between the second and booster doses (77.0 days vs. 180.0 days) and the 1st and 2nd doses (73.0 days vs. 78.0 days) were shorter than those in our study. Although the IgG level was significantly elevated after the third-dose vaccination in the UK COV-BOOST trial, as well as in our study, factors such as groups, ethnicities, study population age, and intervals between each vaccination should also be considered as the causes of differences.

While most participants experienced mild adverse reactions within 3 days, in our study, severe adverse reactions (1.3%) were recorded in less than 5% of the participants, similar to that reported in a previous study on safety after the BNT162b booster injection primed with two doses of BNT162b2 (8). The applicability of BNT162b as a booster to individuals in the age group of 20–30 years was demonstrated to be safe and tolerable by our results. Although approximately three-quarters of our participants (66.0%) took antipyretics, 36.6% of them took prophylactically. Regarding the order of severity, most participants (76.4%) reported that the adverse reactions were the most severe after the first dose of ChAdOx1 nCov-19 among all three doses of vaccines and most of the workers (77.8%) reported least severity after the booster dose compared with those after the 1st and 2nd ChAdOx1 nCoV-19 doses, revealing less safety concerns in this regard.

The SARS-CoV-2 antibody assays targeting total and IgG antibodies showed almost perfect agreement (kappa = 0.919), whereas their agreement with neutralizing antibodies was relatively weak. Concordant with the results, correlation between the total and IgG antibodies was the highest among the three SARS-CoV-2 assays (ρ = 0.955). A previous study comparing 12 commercial SARS-CoV-2 antibody assays showed that the agreement rate and correlation between Roche total and Abbott IgG assays (95.7%; ρ = 0.883) was higher than those with the GenScript neutralizing antibody assay (89.2%; ρ = 0.672 for Roche total and 87.8%; ρ = 0.628 for Abbott IgG) (28). Another study comparing five SARS-CoV-2 antibody assays also showed a high agreement rate between Roche total and Abbott IgG (98.6%) (12). Based on our results, correlations of the three antibody assays with Covi-FERON were found to be weak or negligible. Consistent with our results, cellular responses did not correlate well with humoral responses in the UK COV-BOOST trial, especially for neutralizing tests (8). The characteristics of humoral and cellular immunity, such as different waning pace, might be the cause of this phenomenon.

This study had some limitations. First, the performance of the IGRA test was not verified in this study. According to the manufacturer’s internal data, its specificity was 94.1% (2/34) when testing 34 healthy individuals before vaccination. Second, we could not determine whether the 11 IGRA-negative results in this study were true negatives, due to decreased cellular response 24 days after booster vaccination, or false negatives. More studies, including serial evaluation immediately after vaccination, might provide further information about cellular immunity and the usefulness of this IGRA kit. Finally, healthcare workers as the study population can be both a strength and weakness. For generalization, further assessment in children and older participants would be required in the future.

In conclusion, a third dose of BNT162b2 after two doses of ChAdOx1 nCoV-19 induced 100% positivity based on three representative SARS-CoV-2 antibody assays targeting total, IgG, and neutralizing antibodies. Regarding INF-γ release determined using Covi-FERON, 92.7% positivity was observed. There was no safety concern for the participants. Agreement among the applied antibody assays was substantial or almost perfect. Meanwhile, the correlations of the three antibody assays with Covi-FERON were weak or negligible, indicating differences in humoral and cellular responses. This is the first report of longitudinal antibody titer changes during a 10-month study period before and after three doses of vaccines (ChAdOx1 nCoV-19, ChAdOx1 nCoV-19, and BNT162b2) based on three representative SARS-CoV-2 antibody assays and Covi-FERON among healthy healthcare workers. Furthermore, this study included information about humoral and cellular responses in the East Asian population. The results of our assessment could facilitate the establishment of criteria for booster vaccination strategies and eventually contribute to the control of the spread of COVID-19.

## Data Availability Statement

The datasets presented in this study can be found in online repositories. The names of the repository/repositories and accession number(s) can be found below: HARVARD Dataverse, https://doi.org/10.7910/DVN/LC9AEZ.

## Ethics Statement

The studies involving human participants were reviewed and approved by Institutional Review Board of Hallym University Dongtan Sacred Heart Hospital (HDT 2021-02-007-004) and Institutional Review Board of Hallym University Kangnam Sacred Heart Hospital (HKS 2021-02-030-003). The patients/participants provided their written informed consent to participate in this study.

## Author Contributions

SJ, NL, and HSK conceived and designed the experiments. EJC, JH, MJP, and WS coordinated the study. SKL collected the samples and performed the experiments. SJ and HSK performed data analysis and wrote the manuscript. All authors contributed to the manuscript and approved the submitted version.

## Conflict of Interest

The authors declare that the research was conducted in the absence of any commercial or financial relationships that could be construed as a potential conflict of interest.

## Publisher’s Note

All claims expressed in this article are solely those of the authors and do not necessarily represent those of their affiliated organizations, or those of the publisher, the editors and the reviewers. Any product that may be evaluated in this article, or claim that may be made by its manufacturer, is not guaranteed or endorsed by the publisher.
